# Cesarean Scar Ectopic Pregnancy: A Rare Case

**DOI:** 10.7759/cureus.54920

**Published:** 2024-02-26

**Authors:** Jalormy S Joshi, Jyotsana Potdar, Amardeep Shanoo, Nainita Patel

**Affiliations:** 1 Obstetrics and Gynaecology, Jawaharlal Nehru Medical College, Datta Meghe Institute of Higher Education and Research, Wardha, IND; 2 Obstetrics and Gynaecology, Jawaharlal Nehru Medical College, Datta Meghe Institute of Medical Science (Deemed University), Wardha, IND; 3 Obstetrics and Gynaecology, Jawaharlal Nehru Medical College, Datta Meghe Institute of Higher Education and Research, WARDHA, IND

**Keywords:** diagnostic challenge, ectopic, doppler ultrasonography, caesarean section, caesarean scar ectopic

## Abstract

Cesarean scar ectopic pregnancy is one of the rarest of all ectopic pregnancies. Cesarean scar ectopic can occur after previous uterine manipulation, in vitro fertilization, hysterotomy, etc. With the increasing number of cesarean sections, the incidence of cesarean scar ectopics has increased worldwide. A high degree of suspicion over the occurrence of ectopic pregnancy after a cesarean section should be maintained by all healthcare workers. Timely diagnosis and treatment according to the presentation of an individual is of utmost importance. Here, we present a case of a 24-year-old second gravida with nine weeks of amenorrhea and a previous cesarean section presenting with the possibility of a scar ectopic, initially managed with medical management, followed by a planned laparotomy.

## Introduction

Cesarean scar ectopic pregnancy (CSEP) is the rarest of all types of ectopic pregnancies. When the implantation of a blastocyst occurs on a scar from a previous cesarean section, cesarean scar ectopic pregnancy occurs [1]. Over the past few decades, the prevalence of this scar ectopic has increased dramatically, reaching about 21% globally [2].

Its occurrence has been rising in number along with the global increase in cesarean sections (C-sections). Complications, including severe hemorrhage, abnormal placentation, uterine rupture, and significant morbidity and mortality for the mother, can arise from it. As a result, it becomes essential to precisely diagnose scar ectopic pregnancies early on to prevent complications and maintain future fertility [3]. Transvaginal ultrasonography (USG) is primarily used to make an early diagnosis, which is then verified by MRI to aid in timely management.

Treatment modalities are decided based on the presentation of the case. Medical management with methotrexate or surgical removal of an ectopic can be done. Some women have been managed expectantly also [4]. In addition to surgical excision with a laparoscopy, hysteroscopy, or laparotomy, the ectopic scar can also be removed by vacuum aspiration [5].

## Case presentation

A 24-year-old female, second gravida with nine weeks of amenorrhea and a previous cesarean section, came into the outpatient department of our hospital with the chief complaint of pain in the lower abdomen localized to the area where the previous scar of the lower segment cesarean section (LSCS) was present. The patient had checked for pregnancy at home with a urine pregnancy test (UPT), which was positive. The patient has been experiencing intermittent spotting per vaginum for the last month. The spotting was infrequent in occurrence, hardly soaking one pad a day, and she did not take any consultation for the same. The patient started having pain at the previous LSC scar for the last seven days, for which she underwent ultrasonography (USG), which suggested the possibility of a scar ectopic.

On physical examination, she was vitally stable, with a pulse rate of 96 beats per minute and a blood pressure of 100/60 mmHg. Pallor was present. On examination, the abdomen was soft with no palpable mass, but tenderness at the scar site was present. On per speculum examination, the cervical os was closed, and there was minimal blood in the vaginal canal. On per vaginal examination, the cervix was posterior with closed os, bilateral fornices were free, but there was minimal tenderness present on cervical motion. On investigations, her routine blood investigations, including a complete blood profile, urine analysis, and liver and renal function tests, were normal. Her beta-human chorionic gonadotropin (HCG) level was 332.01 mIU/mL on admission. The transvaginal USG was suggestive of a large, heterogeneous, round-to-oval-shaped mass showing high peripheral vascularity in the lower uterine segment measuring 6.7x6x5 cm, with the possibility of scar ectopic. There was no evidence of any gestational sac noted. MRI pelvis could not be done, as the patient was not able to afford it.

**Figure 1:**
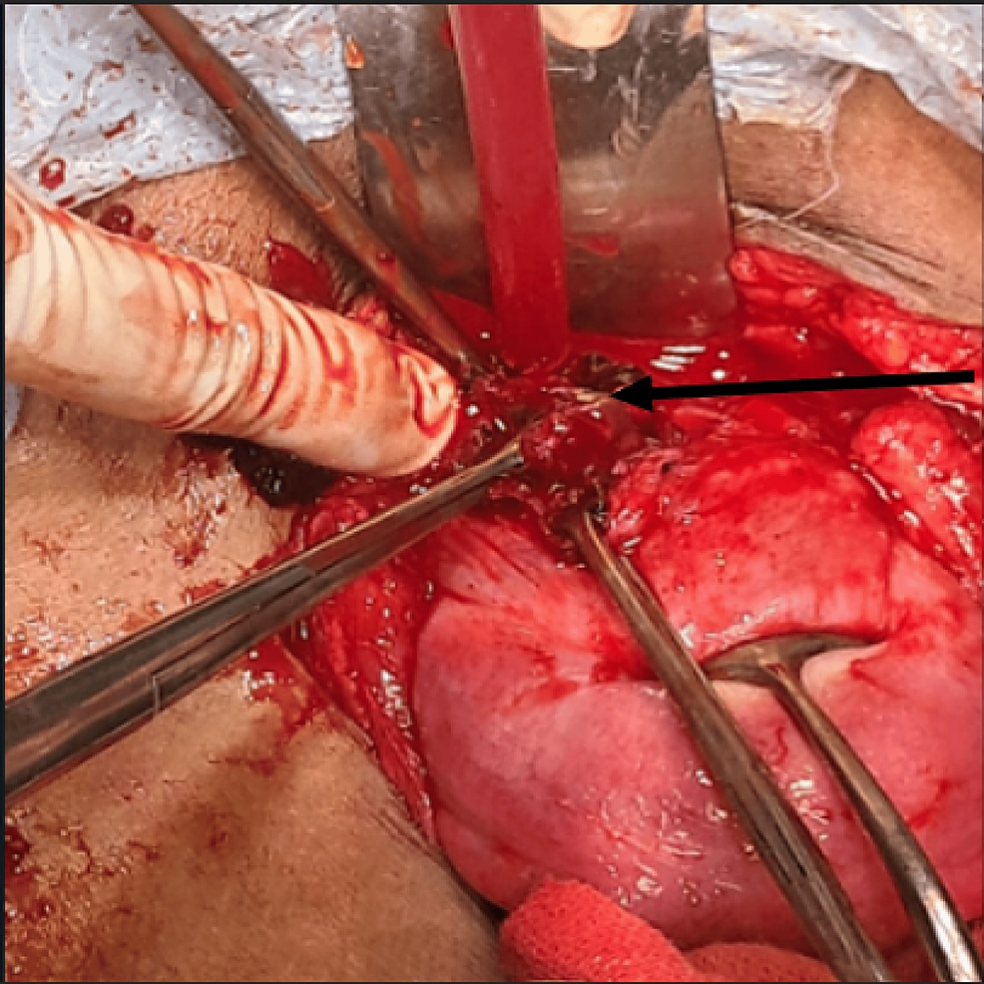
Intra-operative picture of the cesarean scar ectopic showing the implantation at the previous lscs scar (black arrow)

**Figure 2:**
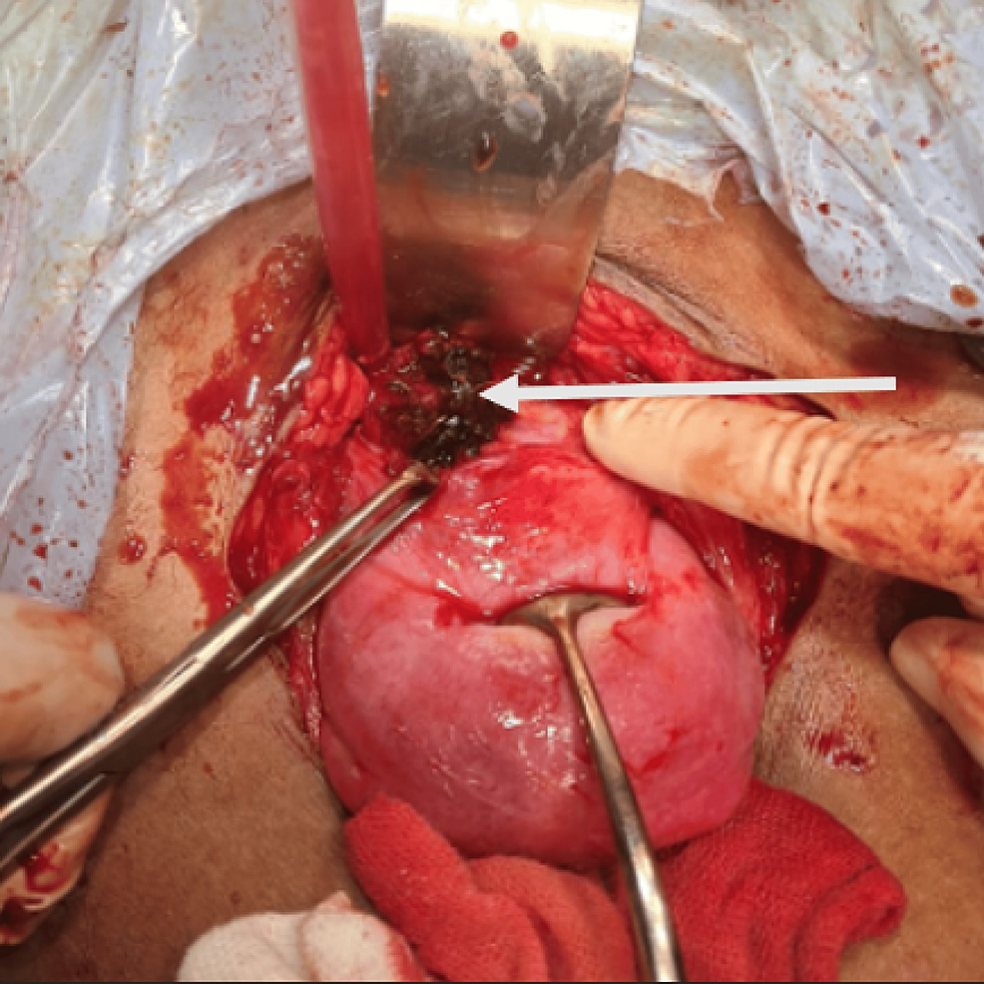
Intra-operative image of the cesarean scar ectopic showing the products of conception (white arrow)

As the patient was vitally stable, the option of medical management was chosen and the patient was treated with Inj. methotrexate 50 mg/m^2^ IM single dose. Beta (B)-HCG was repeated on day 5 and day 7, which were 296.46 mIU/ml and 220.34 mIU/ml, respectively. But, as the decline in B-HCG was suboptimal, the patient was planned for laparotomy. A Pfannenstiel incision was taken along the previous scar to open the abdomen and the uterus was identified. There was a soft and vascular mass present at the previous lscs scar. Myometrium was absent at the implantation site and the mass was just covered by a fold of peritoneum. A transverse incision was taken and the product of conception was gently taken out. The uterine cavity was communicating with scar ectopic pregnancy. The edges of scar tissue were excised, freshened, and closed. A diagnostic hysteroscopy followed by gentle curettage was done. Histopathological examination of the products of conception was done, which was diagnostic of scar ectopic pregnancy. Here, Figure 1 shows the implantation of ectopic at the previous caesarian section scar, and Figure 2 shows products of conception within that scar ectopic pregnancy (Figures 1, 2).

The patient withstood the procedure well and followed up till serum B-HCG levels came back to a non-pregnant level. She was counseled not to conceive for at least three years.

## Discussion

Though the pathophysiology of cesarean scar pregnancy is unclear, there is a theory that the lower uterine segment anteriorly is poorly vascularized, which may hinder healing post-cesarean section in some women and make them more susceptible to developing minor dehiscent tracts and defects where trophoblasts may implant [6,7].

Vaginal bleeding and abdominal pain are the main clinical features of this patient. Heavy vaginal bleeding with acute abdominal pain is concerning for an impending rupture. If the patient comes with a rupture of the CSP through the myometrium, hemodynamic instability will be present.

While previous cesarean sections are the most common cause of myometrial abnormalities, other uterine procedures, such as hysteroscopy, dilatation and curettage (D&C), metroplasty, and myomectomy, have also been linked to scar pregnancies [7,8]. The number of cesarean sections done before the occurrence of CSEP has no independent risk factor for developing cesarean scar pregnancy [4].

Combined transvaginal ultrasonography and color Doppler ultrasonography is the main diagnostic technique for suspected CSEP [4], and it is also the most successful one, with an 86.4% sensitivity rate [9].

A functional trophoblast seen on color Doppler imaging and a gestational sac within a visible myometrial defect positioned anteriorly, at the level of the internal os, are recognized diagnostic criteria for a cesarean section scar pregnancy [10]. It consists of an unoccupied cervical canal and an empty uterus with a clearly defined endometrium.

Acceptable treatment options for CSEPs are dependent on the location of the CSEP, gestational age, and clinical presentation [11]. Traditionally, ectopic pregnancies have been treated with methotrexate if the B-HCG value was less than 5000 mIU/ml. For stable patients, expectant management with uterine artery embolization is also an alternative option, but the patients need to be closely monitored to prevent devastating outcomes like uterine rupture. The most effective treatment option is surgery, which is 96% successful in removing the gestation and repairing the defect of the uterus with conservation of future fertility [9,10]. Surgical excision can be accomplished by vacuum aspiration, hysteroscopy, laparoscopy, or laparotomy [5], which depends on the site of the implantation and the experience of the surgeon, among other factors.

## Conclusions

A cesarean-section ectopic pregnancy occurs when the implantation of the embryo is in the myometrium of the previous cesarean-section scar. Possible outcomes are serious maternal morbidity and mortality, including life-threatening hemorrhage, and uterine rupture, leading to the need for a hysterectomy. In all patients with a previous cesarean section, a high degree of suspicion should be maintained by all healthcare workers for scar ectopic pregnancy. The timely diagnosis and early detection of CSP using imaging are essential for preventing maternal morbidity and death. It is advisable to think about terminating the pregnancy in the first trimester and to individualize the course of treatment.
